# Supplementary material for: Utilizing an Endogenous Progesterone Receptor Reporter Gene for Drug Screening and Mechanistic Study in Endometrial Cancer
Source: Cancers (Basel). 2022 Oct 6;14(19):4883. doi: 10.3390/cancers14194883 (PMC9561963; doi:10.3390/cancers14194883)

Utilizing an endogenous PR reporter gene for drug screening and mechanistic discovery in endometrial cancer

Yiyang Li, Wei Zhou, Xiangbing Meng, Sarina D. Murray, Long Li, Abby Fronk, Vanessa J. Lazaro-Camp, Kuo-kuang Wen, Meng Wu, Adam Dupuy, Kimberly Leslie, Shujie Yang

Supplemental Table S1. Timeline of building a PR reporter gene and its application

1	**Synthesize sgRNA**:	Find PAM-NGG sequence at the end of exon 8 and synthesize sgRNA for CRISPR-Cas9 to generate the double strand break (DSB).
2	**Generate DSB**:	Transfect CRISPR-Cas9-sgRNA into Ishikawa and Hec50 cells to generate the double strand break (DSB).
3	**Detect DSB**:	Detect the break in PGR exon 8 in both cells with Alt-R assay.
4	**Synthesize reporter gene**:	Design and synthesize PR reporter gene containing mCherry and hygromycin, subclone it into the pL253-TK vector, and DNA sequence the vector to validate the reporter gene.
5	**Co-transfection**:	Co-transfect CRISPR-Cas9-sgRNA targeting PR exon 8 and the PR reporter gene vector into Ishikawa and Hec50 cells.
6	**Dual selection**:	Selection of the transfected cells by puromycin and ganciclovir treatment.
7	**Screen right clone**:	Grow single clones and validate the right clone with correct insertion of the PR reporter gene using junction PCR.
8	**Stimulate target gene and reporter gene**:	Grow the PR reporter gene expressing clones and stimulate cells with romidepsin to induce PR and mCherry expression.
9	**Validate reporter gene**:	Validate the correlation of mCherry and PR expression after romidepsin stimulation using qPCR and western blotting.
**10**	**Application-Throughput screening to explore PR inducers**:	Grow reporter gene expressing cells, treat with drugs from drug library and cherry-pick cells with high mCherry signal.
**11**	**Application-Screen PR repressors using GeCKO library**:	Grow reporter gene expressing cells, transduce lentivirus from GeCKO library, followed by flow cytometry to cherry-pick cells with high mCherry signal. Validation of PR induction using qPCR. Each individual GeCKO clone identity revealed by Sanger sequencing of the PCR amplicon expand the sgRNA sequence.

Supplemental Table S2. Primers for generation and confirmation of PR reporter gene

Primer Name	Forward Primer (F)	Reverse Primer (R)
PGR sgRNA	CACCGACCCAAGATATTGGCAGGGA	AAACTCCCTGCCAATATCTTGGGTC
DSB confirmation primer	(In7-F1) CTGGAATAGGGATGGGAAGAAG	(Ex8-R1) GACACAAAGGGAGTTACCTAGAA
Upstream recombination arm	(F0) TGTGTCTAGGGAGCTGGAATA	(sB1) TTCACCATCCCTGCCAATATC
Reporter gene confirmation primer for hygromycin	(F0) TGTGTCTAGGGAGCTGGAATA	(mHyB2) CCTCCTACATCGAAGCTGAAAG
downstream recombination arm	(mCheF5) GACGGCGAGTTCATCTACAA	(B5) CCTCCTCTCCCTTTCTTCTTTC
Reporter gene confirmation primer for mCherry	(F4) GGTCAGGATTATGAGGTCTTGAG	(B5) CCTCCTCTCCCTTTCTTCTTTC

Supplemental Table S3. Primers for qPCR:

Primer Name	Forward Primer (F)	Reverse Primer (R)
PRAB	ATGTGGCAGATCCCACAGGAGTTT	ACTGGGTTTGACTTCGTAGCCCTT
PRB	TCACCAGCTCTTGGTGCCTGTTT	TCCCGGAGCTGTCTCCAACCTT
PAEP	GAGATCGTTCTGCACAGATGG	CGTTCGCCACCGTATAGTTGAT
FOXO1	TCGTCATAATCTGTCCCTACACA	CGGCTTCGGCTCTTAGCAAA
P21	TGTCCGTCAGAACCCATGC	AAAGTCGAAGTTCCATCGCTC
Myc	GGCTCCTGGCAAAAGGTCA	CTGCGTAGTTGTGCTGATGT
Cyclin D1	CAATGACCCCGCACGATTTC	CATGGAGGGCGGATTGGAA
APH1A	GGTGGTTGGGAGTCACCTACT	GCGCTGAATACTTCGGAGGG
GLUD2	CCCTGCAACCATGTGCTGA	GCTGTAACGGATACCTCCCTTG
PHF13	CATCCCTTATCCGAAGGAGGA	GGACGCGATGTCTGAGAAACC
SCRN1	CTGCCGAGATAGAAGCCTTGC	ACGATCCACAATCAGATATGCAC
SGPP2	ACTGGAAAAGAGACTGATCGCT	CCTGCTGAGACACACCAAGG
TBC1D2B	CCCAGGGCATCCAAATTCCAT	AGGACGGAAAGAAGACATCGAA
BCAS4	AGATGGTTGGACACCACGTC	GCAGGTGACTTCTGTAATCCCA
SOX9	AGCGAACGCACATCAAGAC	CTGTAGGCGATCTGTTGGGG

Supplemental Table S4. sgRNA sequence for knock out target gene using CRISPR-Cas9 technique

Primer Name	sgRNA Sequence
APH1A-1	TCACCAGTCCCGATGTCAGT
APH1A-2	CCATGGATCCCAACCACACC
APH1A-3	TTAGCATCGCTGAGTGAGGA
SGPP2-1	CCATATGATGATCAATCTTC
SGPP2-2	GGGCCACTTCAAGACATCCT
SGPP2-3	AAGAGACTGATCGCTGAATA
SETDB1-1	ACTTCGGCATTTCATCGATG
SETDB1-2	TGAGGAACTGGGTATCTCTA
SETDB1-3	CAGAACTCCAAAAGACCAGA
SOX9-1	CGTGTTCTCGGTGTCCGAGC
SOX9-2	CATGAAGATGACCGACGAGC
SOX9-3	TTCACCGACTTCCTCCGCCG

Supplemental Figure S1. Multiple PR reporter clones confirmed by PCR of P5 primer pairs and Western blotting by mCherry antibody. (**A**) The EM25 PR reporter clone is an ECC1 clone derived from PR reporter plasmids only with M-cherry reporter. The E28 clone is an ECC1 cells derived from reporter plasmids with both M-cherry and Hygromycin resistant gene. The I12L PR reporter clone is derived from Ishikawa cells transfected with PR reporter plasmids with both M-cherry and Hygromycin resistant gene. (**B**) mCherry expression was induced by 20nM Romidpson in representative PR reporter clones I12L, EM25 and E28. treated for 8 hrs,16hrs, 24hrs and 48hrs.

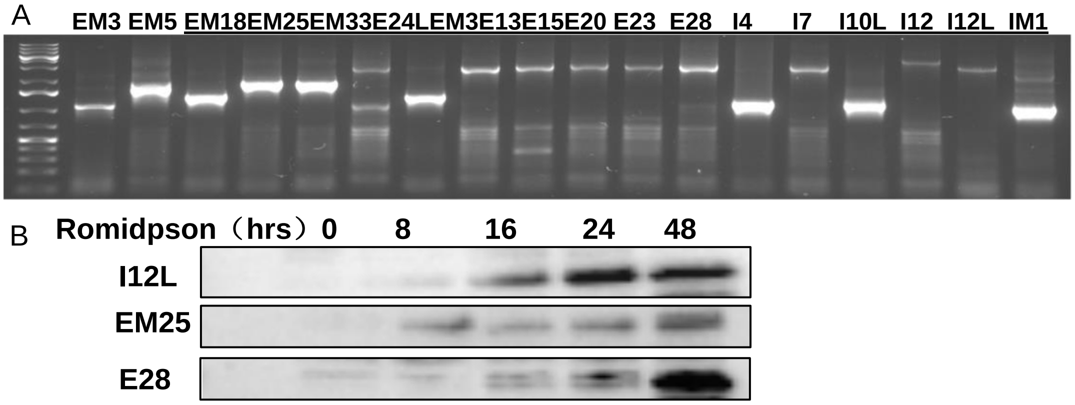

Supplemental Figure S2. mCherry signal of Ishikawa cells expressing PR reporter gene increased when treated with the increased dose of carfilzomib and daunorubicin. Ishikawa cells were grown in 3D culture and treated with different dose of carfilzomib and daunorubicin. mCherry signals were captured by the Operetta High Content Screening System (PE).

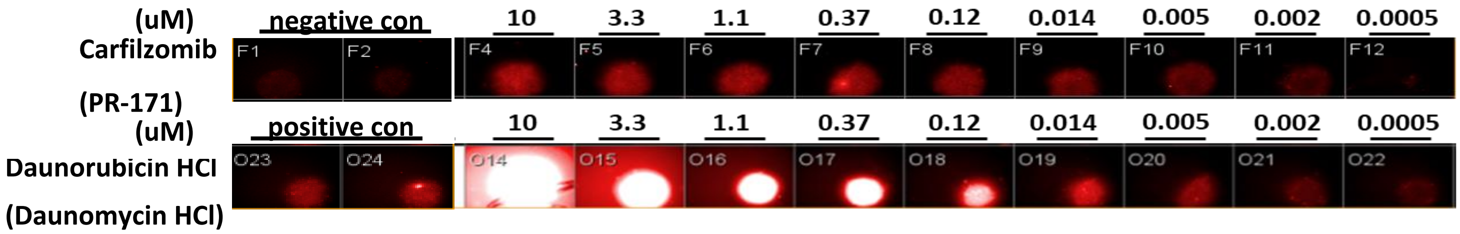

Supplemental Figure S3A. Cell proliferation assay of Ishikawa cells in response to different dose to indicated drugs. Ishikawa cells were treated with the different dose of drugs for 72hr and followed by staining with crystal violet.

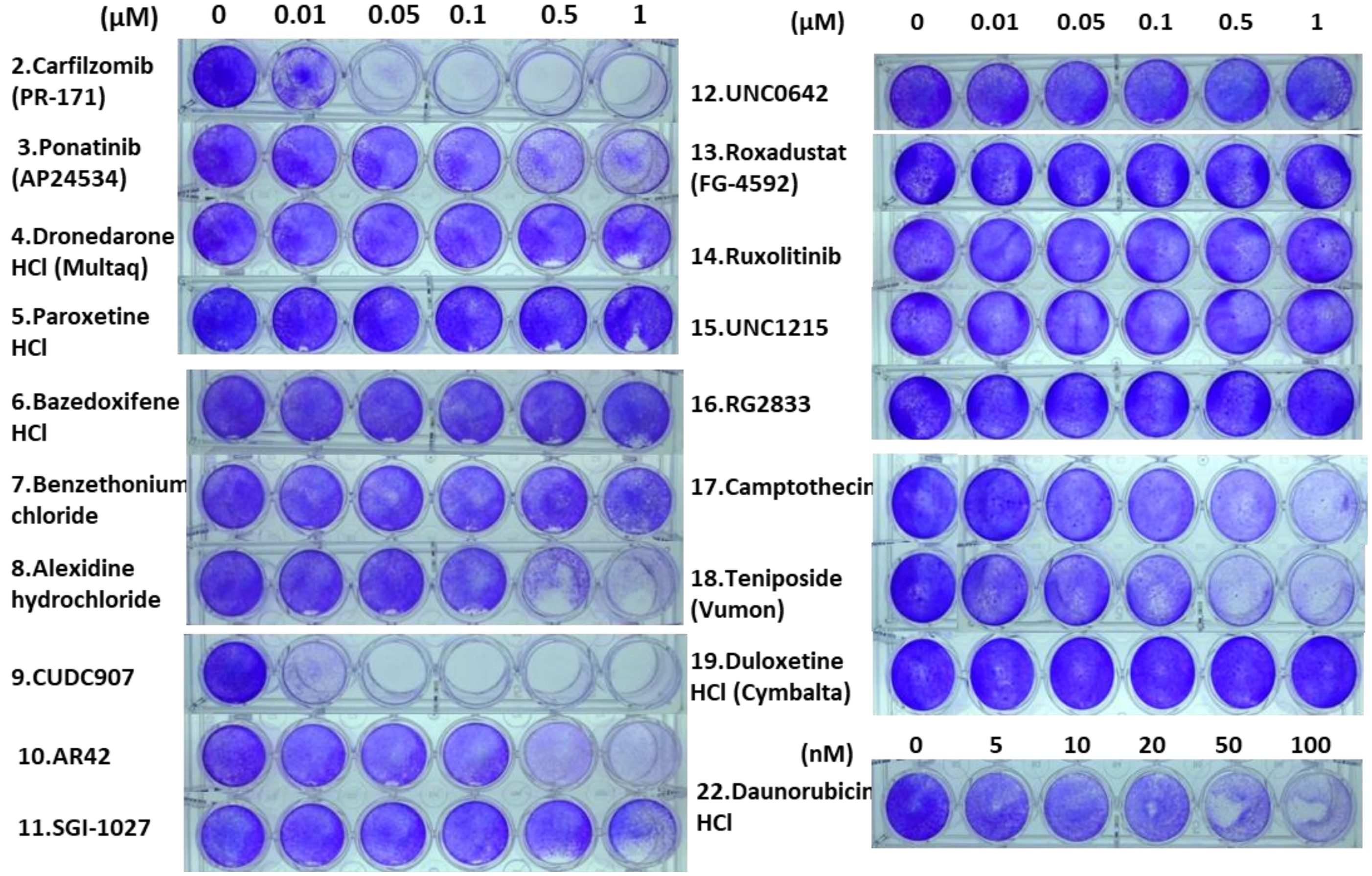

Supplemental Figures S3B. Western blotting of PR and its downstream genes after the treatment with the 5 top-picked drugs, Carfilzomib [100nM], CUDC-907 (20nM), AR-42 (500nM), romidepsin (20nM), daunorubicin (100nM) for 24hr in KLE cells.

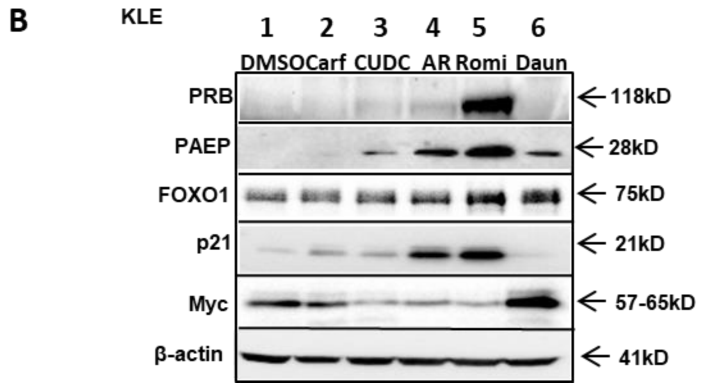

Supplemental Figure S4. mRNA expression of PR and target genes. (**A**) mRNA expression of PR and potential PR repressors in the representative clones from GeCKO library. (**B**) mRNA expression of PRB in SGPP2 and GLUD2 knockout clones of Ishikawa and ECC1 cello lines.

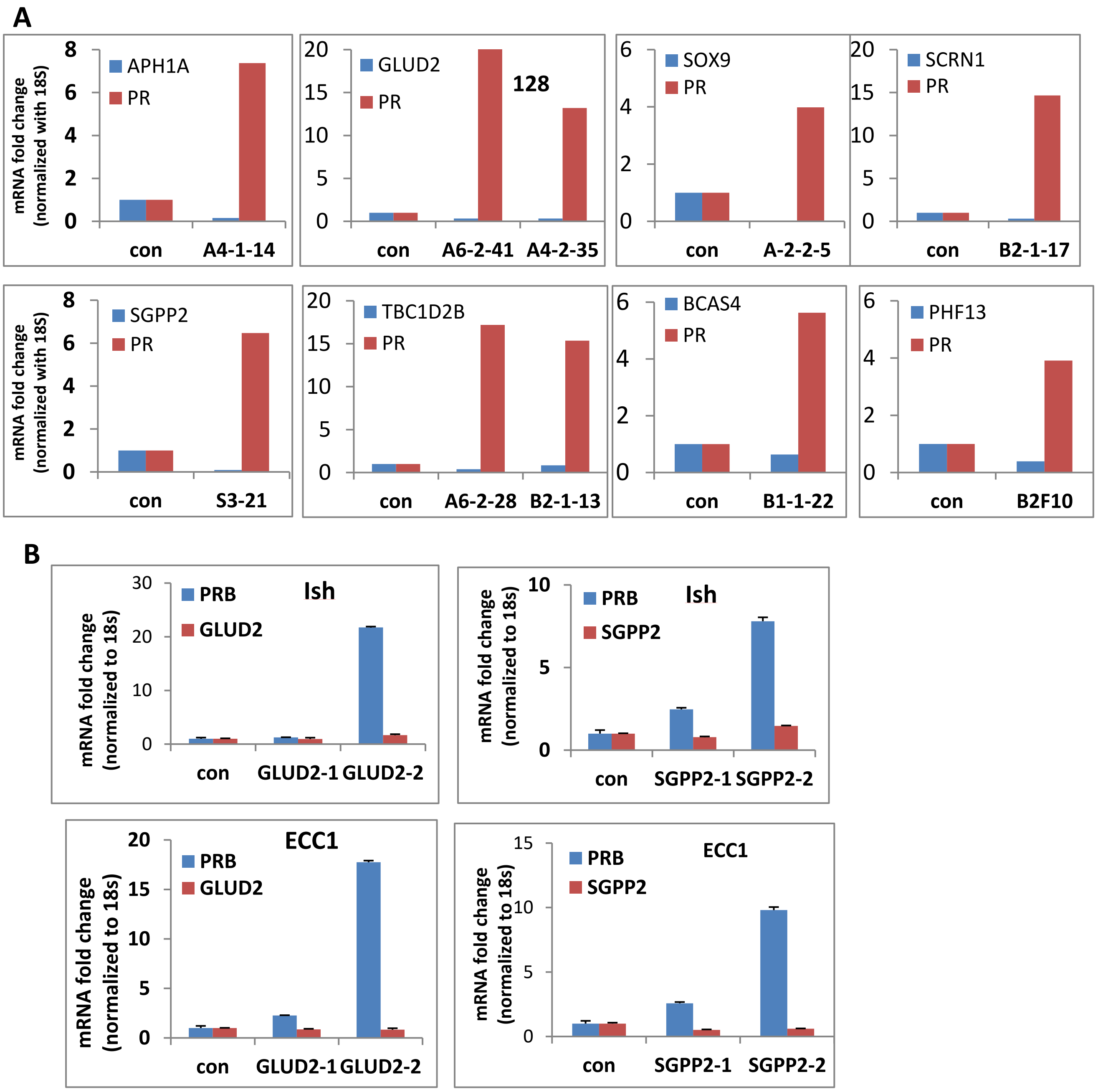

Supplemental Figure S5. High expression of APH1A, GDH2, SETDB1, SOX9 and BCAS4 associated with worse survival in endometrial cancer. Correlation of endometrial cancer patient survival with mRNA expression of indicated genes were generated using endometrial cancer TCGA data which is available in The Human Protein Atlas website (https://www.proteinatlas.org/).

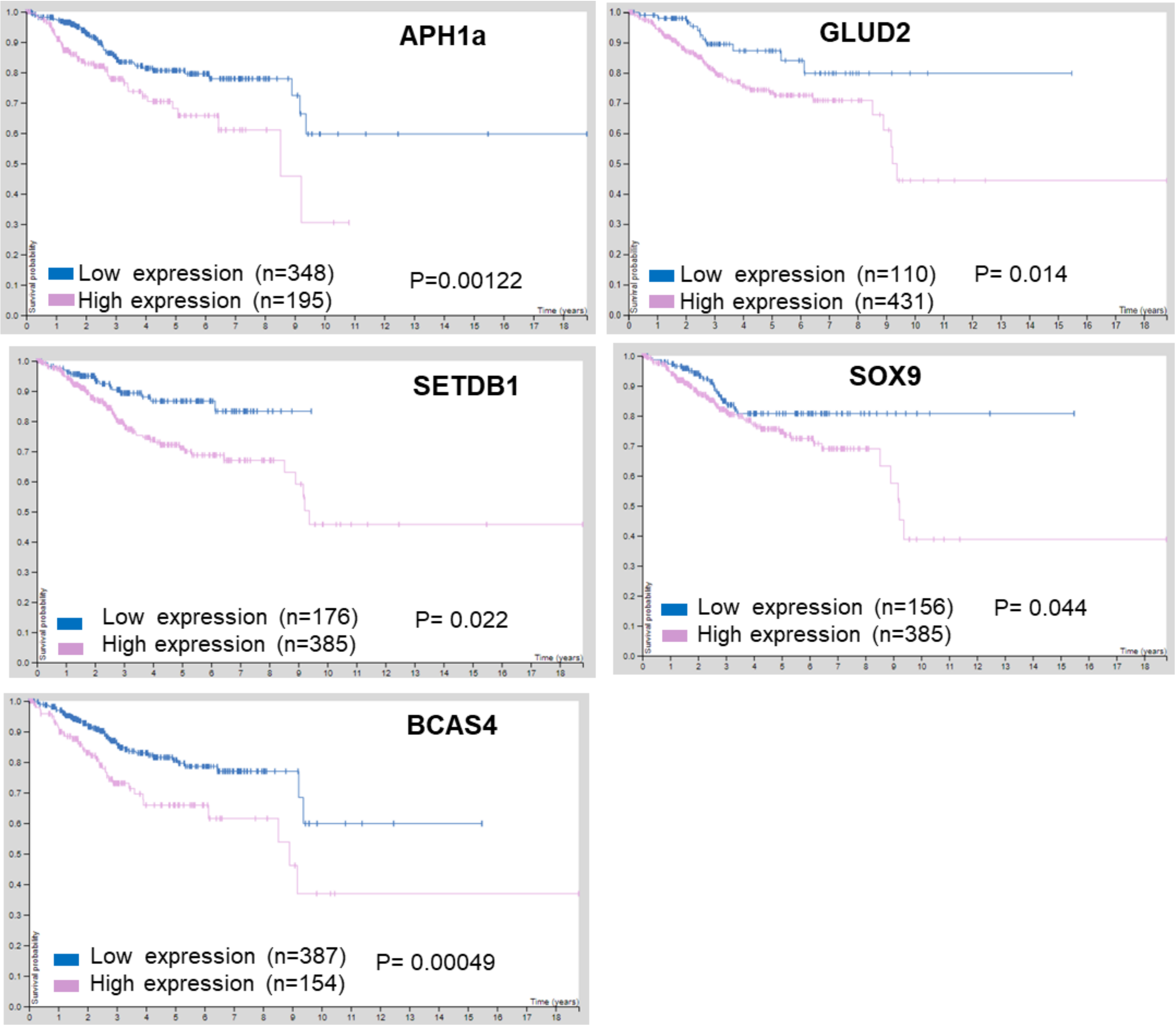

Supplemental Figure S6. The whole blot (uncropped blots) showing all the bands with all molecular weight markers on the Western Original WB blots for Fig. 2B, 3E, 3G, 5A, 5B.

Figure 2B

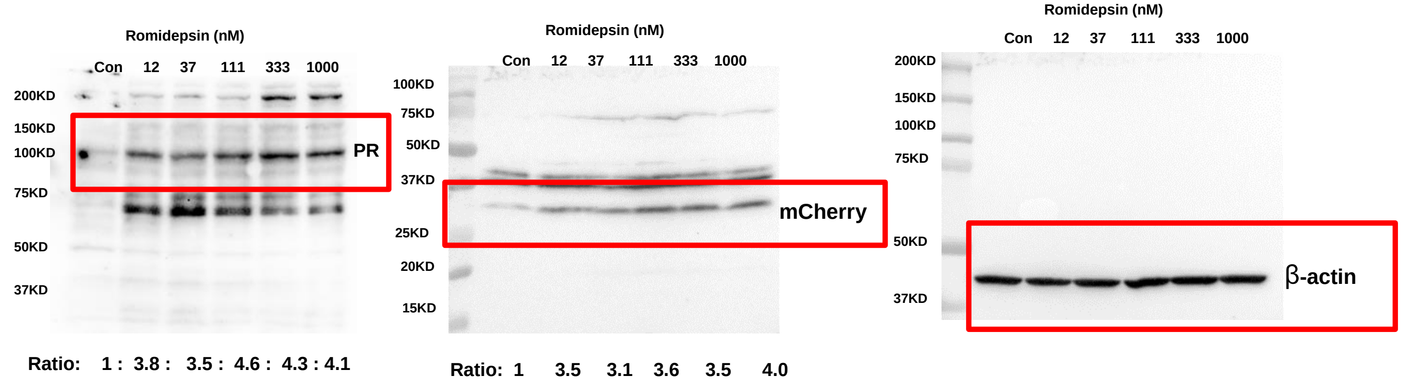

Figure 3E

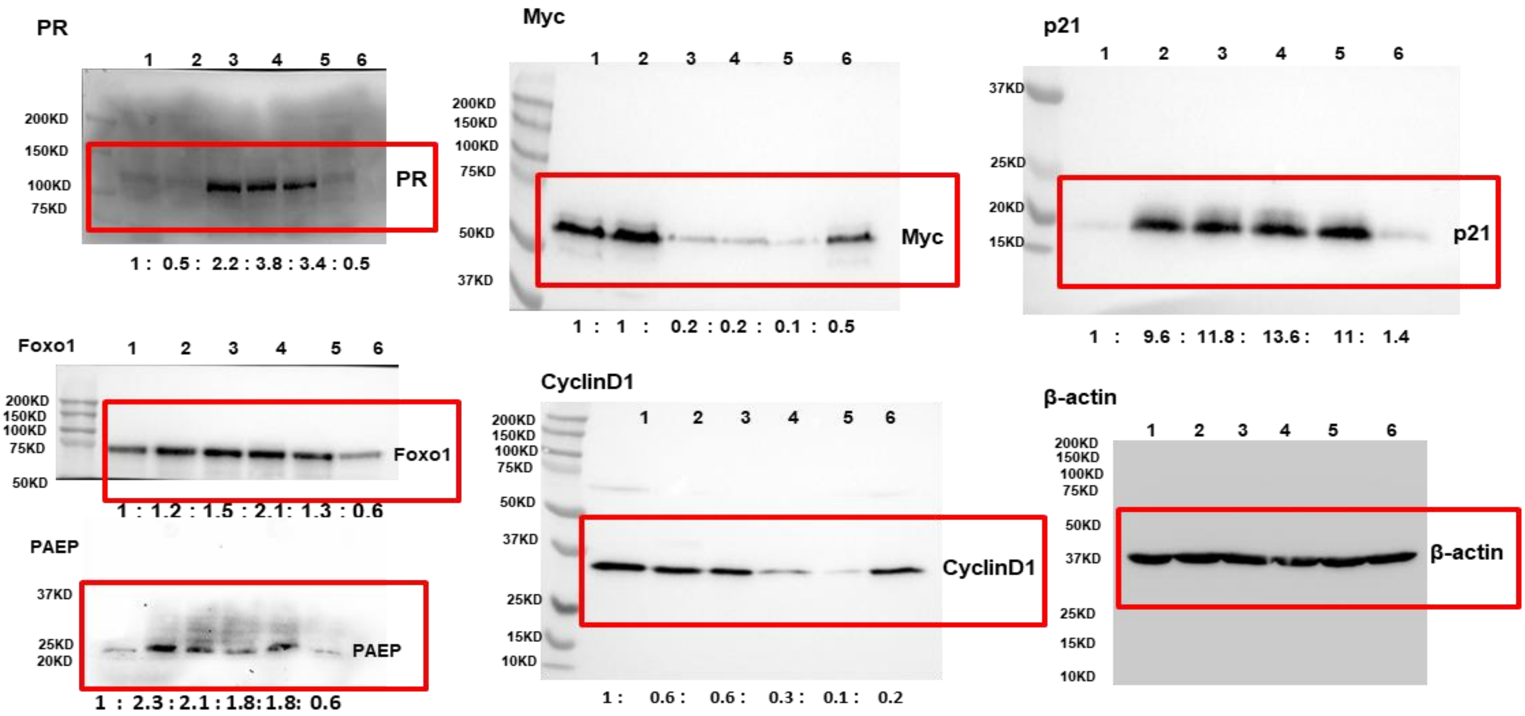

Figure 3G

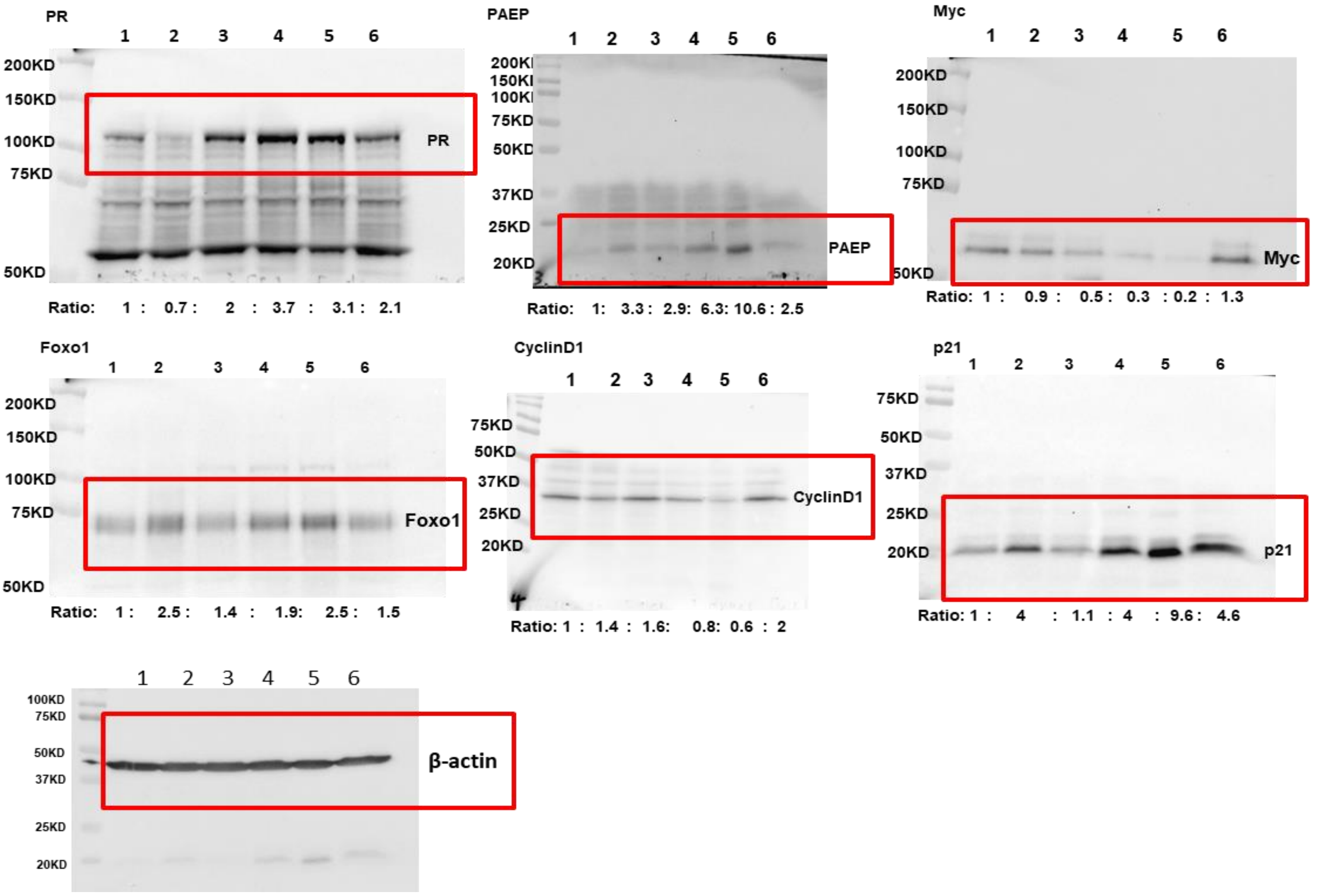

Figure 5

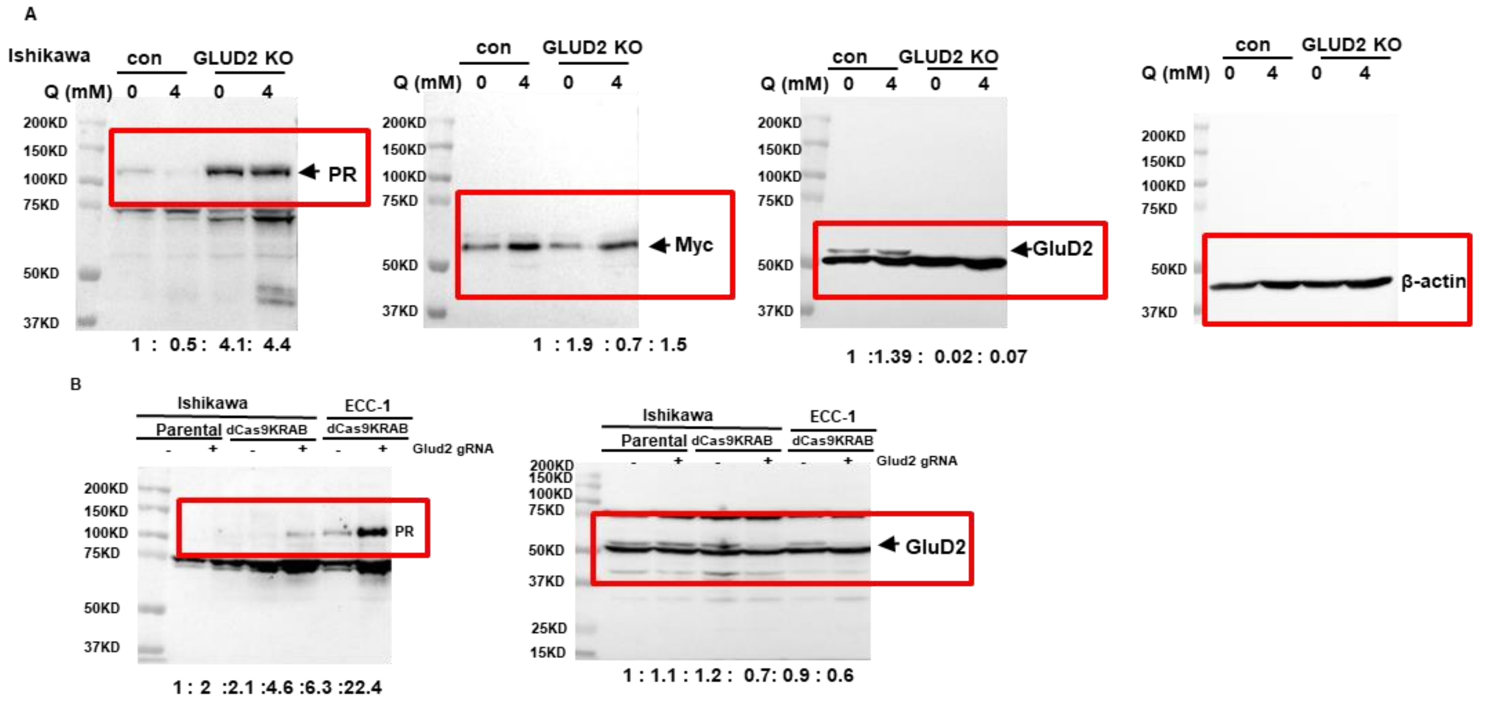

Supplement: Supplementary file 1 [file cancers-14-04883-s001.zip › cancers-1841713-supplementary.pdf]
